# Elevated serum levels of aminotransferases in relation to unhealthy foods intake: Tehran lipid and glucose study

**DOI:** 10.1186/s12902-019-0437-5

**Published:** 2019-10-15

**Authors:** Parvin Mirmiran, Zahra Gaeini, Zahra Bahadoran, Fereidoun Azizi

**Affiliations:** 1 Department of Clinical Nutrition and Dietetics, Faculty of Nutrition Sciences and Food Technology, National Nutrition and Food Technology Research Institute, Shahid Beheshti University of Medical Sciences, Tehran, Iran; 2 Nutrition and Endocrine Research Center, Research Institute for Endocrine Sciences, Shahid Beheshti University of Medical Sciences, No. 24, Shahid-Erabi St., Yeman St., Velenjak, P.O. Box: 19395-4763, Tehran, Iran; 3 Endocrine Research Center, Research Institute for Endocrine Sciences, Shahid Beheshti University of Medical Sciences, Tehran, Iran

**Keywords:** Unhealthy foods, Fast foods, Soft drinks, Snacks, Aminotransferases, Liver enzymes

## Abstract

**Background:**

Abnormal levels of liver enzymes, particularly aminotransferases, are prognostic features of non-alcoholic fatty liver disease (NAFLD). Considering the important role of dietary intakes in development of NAFLD, we aimed to determine possible association of unhealthy foods (fast foods, soft drinks, sweet and salty snacks) consumption with elevated levels of aminotransferases.

**Methods:**

This cross-sectional study was conducted within the framework of sixth phase of the Tehran Lipid and Glucose Study (2014–2017), on 187 adult men and 249 adult women (19–70 y). Usual intakes of unhealthy foods (kcal/week) were measured using a validated semi-quantitative 147-items food frequency questionnaire. Serum levels of alanine aminotransferase (ALT), aspartate aminotransferase (AST) were measured. Multivariable logistic regression models were used to estimate the odds of elevated aminotransferases in each tertile of energy-dense unhealthy foods.

**Results:**

Mean age of participants was 44.44 ± 15.09 years, 43% of participants were men. Higher consumption of fast foods (> 11.39% kcal/week) was associated with elevated ALT to AST ratio (OR: 3.27; 95% CI: 1.90–5.63) and elevated ALT (OR: 2.74; 95% CI: 1.57–4.76). Also, each 1 SD increased energy intakes from fast foods was related to increased chance of having elevated ALT and ALT to AST ratio by 35% (OR: 1.35; 95% CI: 1.08–1.68, OR: 1.35; 95% CI: 1.10–1.66, respectively). There was no significant association between consumption of soft drinks, sweet or salty snacks and elevated aminotransferases.

**Conclusions:**

Higher intakes of energy from fast foods seems to be associated with an elevated serum levels of ALT and ALT to AST ratio, as indicators of development of NAFLD.

## Background

Nonalcoholic fatty liver disease (NAFLD), encompassed a large spectrum of conditions from simple hepatocellular steatosis to inflammatory fibrosis, cirrhosis and in some cases hepatocellular carcinoma [1], is associated with increased risk of liver transplantation and all-cause mortality [2]. Epidemiological studies indicate that the global prevalence of NAFLD varies among different population with different ethics, with a higher degree in Asians and Hispanics [3]. The prevalence of NAFLD has been estimated 33.9% in Iran [4], in line with increased prevalence of obesity which has been estimated 31.3% in Iranian population [5], as a major risk factor for NAFLD. With that said, there is a growing interest in regard to NAFLD dietary risk factors, which along with the lack of inconsistent data, necessitates the attention of academic community.

Along with reliable diagnostic methods for NAFLD such as radiologic or histologic features, abnormal liver enzyme levels, particularly aminotransferases, are suggested as prognostic features of liver dysfunction [6]. Alanine aminotransferase (ALT) is the most important surrogate of liver dysfunction [7]; elevated levels of ALT has been shown to be strongly correlated with NAFLD [8–10]. Some reports suggest that serum ALT to aspartate aminotransferase (AST) ratio (ALT/AST) is the most relevant predictor of fatty liver [11], insulin resistance [12] and metabolic syndrome [13, 14]. The accepted upper limit of aminotransferases i.e. 40 U/L, is now under the debate [15–17]. The upper limits of normal ALT in Iranian populations has been estimated lower than cut-offs determined by laboratory manufactures [18–20]. Currently no optimal cut-off value has been defined for liver enzymes to predict NAFLD in population-based studies [21].

Dietary factors have major role in development of NAFLD and its related disorders [22]. Poor dietary patterns such as Western pattern, characterized by high intakes of unhealthy foods with high calorie and poor nutrients (fast foods, soft drinks and snacks), were related to increased risk of metabolic syndrome [23, 24], obesity, cardiovascular disease [25, 26], and NAFLD [27, 28]. Although the association between Western dietary pattern and NAFLD was reported previously, there is limited data on the association between specific components of Western diet, including fast foods, soft drinks and snacks and liver enzyme levels. The aim of the current study was to determine the association of unhealthy foods (fast foods, soft drinks, sweet and salty snacks) with elevated levels of ALT and ALT to AST ratio in the framework of a population-based study.

## Methods

### Study population

In the present study we used data collected from the Tehran Lipid and Glucose Study (TLGS). Briefly, TLGS is a population-based study on a representative sample including 15,005 individuals of residents from district 13 of Tehran [29], which was initiated from 1999 and data collection is ongoing at 3-year intervals [30]. For the current analysis, subjects were excluded from the study if they were under the age of 18, had incomplete data on liver function test (LFT), demographics, anthropometrics, biochemical measurements and dietary intakes in the sixth TLGS examination (2014–2017). Finally, we recruited 436 adult men and women (age ≥ 19 years) into the analysis.

### Anthropometric and demographic measures

Weight of participants was measured using digital scales (Seca, Hamburg, Germany), while the subjects were minimally clothed and without shoes, and reported to the nearest 100 g. Height was measured using a tape meter, in a standing position and without shoes, and recorded to the nearest of 0.5 cm. Body mass index (BMI) calculated as weight (kg) divided by height in square (m^2^). Waist circumference was measured using a tape meter, without any pressure to the body, between the lower border of the ribs and the iliac crest at the widest portion, while the subjects were lightly clothed.

For measurements of systolic (SBP) and diastolic (DBP) blood pressures, we used a standard mercury sphygmomanometer calibrated by the Iranian Institute of Standards and Industrial Researches [31]. Two measurements of blood pressure were taken on the right arm of the participants, with at least a 30-s interval between two measurements, and a 15-min rest before measurement, while they were in a sitting position. Mean of the two measurements was considered as the participant’s blood pressure.

### Biochemical measures

Blood samples was drawn in the fasting state, between 7:00 and 9:00 AM. Serum liver enzymes (ALT and AST) were assayed using enzymatic colorimetric methods. All blood analysis were done using Pars Azmoon kits (Pars Azmoon Inc., Tehran, Iran) and a Selectra 2 auto-analyzer (Vital Scientific, Spankeren, The Netherlands) at the research laboratory of the TLGS. Both inter- and intra-assay coefficients of variations (CVs) were less than 5%.

### Dietary assessment

Dietary assessment of typical food intakes over the previous year was done using a validated 147-item food frequency questionnaire (FFQ), since this method is simple, cost-effective and time-saving, and is suitable for epidemiological studies [32]. The intake frequency of each food item asked on a daily, weekly, or monthly basis in household measures, and then converted to grams [33]. Participants were questioned about frequency of consuming fast-foods, soft drinks, sweet and salty snacks in the preceding year. The principal dietary exposure of interest was considered weekly energy intakes from fast foods (including pizza, sausage and hamburger), soft drinks (including industrial or cola beverages, industrial fruit juices), sweet-snacks (including biscuits, crackers, cakes, cookies, candies, and chocolates) and salty-snacks (including potato chips, French fries, and puff snacks). Since the Iranian Food Composition Table (FCT) has limited data on nutrient content of raw foods and beverages, we used the US Department of Agriculture’s (USDA) Food Composition Table to analyze foods and beverages for their energy and nutrient contents.

The validity of the food frequency questionnaire has been previously evaluated by comparing dietary values determined from the FFQ with values estimated from the average of twelve 24-h dietary recall surveys, and the reliability has been assessed by comparing dietary values of two FFQs [34].

### Statistical analyses

General characteristics of the participants were compared across median of ALT to AST ratio using independent sample t-test or chi square test, for dichotomous and continues variables, respectively.

Elevated levels of ALT and ALT to AST ratio were considered as the values above the median (12 U/L and 0.62, respectively). Multivariable binominal logistic regression models with adjustment for sex, age (year), and body mass index (BMI) (kg/m^2^) were used to estimate the odds ratio of elevated ALT and ALT to AST ratio in each tertile category of fast-foods, soft drinks, sweet and salty snacks consumption. The first tertile was considered as reference. Total weekly consumption of dietary exposures in the first, second, and third tertiles were < 3.16, 3.16 to 11.38, > 11.38% from kcal/week for fast foods, < 1.50, 1.50 to 5.68, > 5.68% from kcal/week for soft drinks, 3.04 to 13.48, 13.48 to 30.42, > 30.42% from kcal/week for sweet snacks, and < 3.08, 3.08 to 11.66, > 11.66% from kcal/week for salty snacks. In the continues models, the odds of elevated ALT and ALT to AST ratio were calculated per one SD increased intakes of energy from unhealthy foods. To assess the overall trends across increasing tertiles of each unhealthy food group intake and elevated liver enzymes and to determine *P* values for trend, the median of each tertile of fast foods, soft drinks, sweet and salty snacks were used as continues variables in the regression models. Elevated levels of ALT and ALT/AST ratio was used as dependent variables in the regression models. P values obtained from regression models were considered as P values for trend.

All statistical analyses were performed using the Statistical Package for Social Science (version 20; IBM Corp., Armonk, NY, USA). *P*-values < 0.05 being considered significant.

## Results

The mean age of participants was 44.44 ± 15.09 years, and mean BMI was 27.71 ± 5.04 kg/m^2^. Forty-three percent of the participants were men. The mean calorie intake from fast foods, soft drinks, sweet and salty snacks were 254 ± 307 kcal/week, 143 ± 215 kcal/week, 698 ± 758 kcal/week, 292 ± 469 kcal/week, respectively. The mean percentage of calorie intake from fast foods, soft drinks, sweet and salty snacks in a week were 10.77 ± 12.62%, 5.94 ± 8.29%, 29.03 ± 27.78%, 11.85 ± 16.23%, respectively.

Compared with participants who had ALT to AST ratio lower than median, those with the higher values were more likely to be younger (41.33 vs. 47.61 y; *P* = 0.001), more likely to be male (53% vs. 32.4%; *P* = 0.001), had significantly higher weight (79.7 vs. 71.5 kg; *P* = 0.001), waist circumference (93.9 vs. 91.2 cm; *P* = 0.001), and higher level of ALT (22.78 vs. 9.42 U/L; *P* = 0.001) (Table 1).

**Table 1:** General characteristics of participants across median of ALT to AST ratio

Characteristic	ALT/AST < 0.62	ALT/AST ≥ 0.62	*P-Value*
Age (year)	47.61 ± 16.48	41.33 ± 12.76^*^	0.001
Male (%)	32.4	53.0^*^	0.001
Weight (kg)	71.5 ± 14.18	79.7 ± 16.11^*^	0.001
Body mass index (kg/m^2^)	27.25 ± 5.23	28.19 ± 4.82	0.053
Waist circumference (cm)	91.2 ± 13.53	93.9 ± 11.41^*^	0.023
Systolic blood pressure (mm Hg)	114 ± 18.72	113 ± 16.62	0.483
Diastolic blood pressure (mm Hg)	75.4 ± 10.44	76.3 ± 11.09	0.367
Triglyceride (mg/dl)	120 ± 79.2	144 ± 80.2	0.142
High density lipoprotein (mg/dl)	49.82 ± 10.14	45.96 ± 10.95	0.192
Alanine transaminase (U/L)	9.42 ± 7.33	22.78 ± 13.51^*^	0.001
Aspartate transaminase (U/L)	22.80 ± 19.10	21.37 ± 8.99	0.320
Alkaline phosphatase (U/L)	191 ± 114	189 ± 61.8	0.846
gamma-glutamyl transferase (U/L)	25.23 ± 45.29	26.31 ± 19.30	0.749

Data are mean ± SD

^*^Significant differences across median of ALT to AST ratio (*P* < 0.05) (Independent sample t-test was used)

Dietary intakes of participants across median of ALT to AST ratio are shown in Table 2. Participants with elevated ALT to AST ratio had higher energy intakes (2442 vs. 2219 kcal; *P* = 0.001) and energy density (101 vs. 93.7; *P* = 0.001), compared with those who had ALT to AST ratio lower than median. Participants who had elevated ALT to AST ratio had significantly higher dietary intakes of protein (95.6 vs. 82.3; *P* = 0.001), total carbohydrate (358 vs. 332; *P* = 0.041), complex carbohydrate (227 vs. 205; *P* = 0.036), total fat (83.3 vs. 72.8; *P* = 0.001), mono-unsaturated fats (27.82 vs. 24.36; *P* = 0.017), Trans fats (0.15 vs. 0.06; *P* = 0.004), cholesterol (258 vs. 218; *P* = 0.001), total fiber (47.08 vs. 41.93; *P* = 0.014), as well as percentage of energy intakes from fast foods (13.08 vs. 8.54; *P* = 0.001) and soft drinks (6.81 vs. 5.14; *P* = 0.037). There was no significant difference of percentage of energy intakes from sweet and salty snacks between two groups.

**Table 2:** Dietary intakes of participants across median of ALT to AST ratio

Characteristic	ALT/AST < 0.62	ALT/AST ≥ 0.62	*P-Value*
Energy (kcal/d)	2219 ± 771	2442 ± 857^*^	0.005
Protein (g/d)	82.3 ± 30.82	95.6 ± 39.19^*^	0.001
Total carbohydrate (g/d)	332 ± 124	358 ± 133^*^	0.041
Simple sugars (g/d)	127 ± 58.9	131 ± 57.9	0.830
Complex carbohydrate (g/d)	205 ± 77.3	227 ± 90.5^*^	0.036
Total fat (g/d)	72.8 ± 29.04	83.3 ± 36.12^*^	0.001
Saturated fat (g/d)	22.87 ± 10.37	25.93 ± 12.52	0.094
Mono-unsaturated fat (g/d)	24.36 ± 9.96	27.82 ± 12.54^*^	0.017
Poly-unsaturated fat (g/d)	14.90 ± 6.99	16.80 ± 9.06	0.147
Trans fatty acid (g/d)	0.06 ± 0.49	0.15 ± 0.75^*^	0.004
Cholesterol (mg/d)	218 ± 108	258 ± 142^*^	0.001
Total fiber (g/d)	41.93 ± 20.09	47.08 ± 23.06^*^	0.014
Fast food (% from energy/week)	8.54 ± 12.63	13.08 ± 12.32^*^	0.001
Soft drink (% from energy/week)	5.14 ± 7.10	6.81 ± 9.33^*^	0.037
Sweet snacks (% from energy/week)	26.47 ± 25.66	31.34 ± 29.60	0.069
Salty snacks (% from energy/week)	11.89 ± 18.56	11.87 ± 13.72	0.989
Energy density	93.7 ± 21.57	101 ± 23.63^*^	0.001

Data are mean ± SD

^*^Significant differences across median of ALT to AST ratio (*P* < 0.05) (Independent sample t-test was used)

The odds ratio and 95% confidence interval of elevated ALT to AST ratio across tertiles of dietary exposure consumption are presented in Table 3. The chance of elevated ALT to AST ratio (≥0.62) in participants with the highest consumption of fast foods and salty snacks were significantly increased in the crude models (odds ratio (OR): 3.84; 95% confidence interval (CI): 2.35–6.26, and OR: 1.96; 95% CI: 1.23–3.14, respectively). Also, higher consumption of soft drinks in continues and crude model was significantly related to elevate ALT to AST ratio (OR: 1.23; 95% CI: 1.01–1.50). After adjustment for potential confounding variables, the chance of having elevated ALT to AST ratio was 3.20 (95% CI: 1.86–5.51) in the participants who had highest energy intakes from fast foods (> 11.39% of weekly energy intake). Each 1 SD increased in energy intakes of fast foods was related to odds of elevated ALT to AST ratio by 35% (OR: 1.24; 95% CI: 1.01–1.04).

**Table 3:** Odds ratio (95% confidence interval) of elevated ALT to AST ratio across tertiles of unhealthy foods: Tehran Lipid and Glucose Study

	Tertile 1	Tertile 2	Tertile 3	*p* for trend	Continues
Fast-food (%kcal/week)	*< 3.16*	*3.16–11.38*	*> 11.39*		
Crude	1.00	1.81 (1.13–2.92)	3.84 (2.35–6.26)	0.001	1.49 (1.20–1.86)
Adjusted model	1.00	1.64 (0.97–2.77)	3.20 (1.86–5.51)	0.001	1.24 (1.01–1.04)
Soft drink (%kcal/week)	*< 1.50*	*1.50–5.68*	*> 5.69*		
Crude	1.00	1.14 (0.71–1.81)	1.57 (0.98–2.50)	0.060	1.23 (1.01–1.50)
Adjusted model	1.00	0.82 (0.49–1.36)	0.99 (0.58–1.67)	0.905	1.01 (0.98–1.03)
Sweet snack (%kcal/week)	*< 13.48*	*13.48–30.42*	*> 30.43*		
Crude	1.00	0.86 (0.54–1.36)	1.36 (0.86–2.17)	0.195	1.20 (0.98–1.46)
Adjusted model	1.00	0.71 (0.43–1.17)	1.11 (0.66–1.85)	0.832	1.00 (1.00–1.01)
Salty snack (%kcal/week)	*< 3.08*	*3.08–11.66*	*> 11.67*		
Crude	1.00	1.94 (1.21–3.10)	1.96 (1.23–3.14)	0.006	1.00 (0.83–1.20)
Adjusted model	1.00	1.65 (0.99–2.74)	1.36 (0.80–2.32)	0.281	0.99 (0.98–1.00)

Logistic regression model was used (adjusted for sex, age, body mass index, diabetes status)

Elevated ALT to AST ratio was considered as values ≥0.62

Odds and 95% CI of elevated ALT (≥12 U/L) across tertiles of unhealthy foods are shown in Table 4. Higher consumption of fast foods (> 11.39% of weekly energy intake), was associated with elevated ALT in crude model (OR: 2.70; 95% CI: 1.68–4.34) and adjusted model (OR: 2.74; 95% CI: 1.57–4.77). In continues model, more energy intakes of fast foods were significantly associated with elevated ALT in crude model (OR: 1.33; 95% CI: 1.09–1.63). There was no significant association between consumption of sweet or salty snacks and elevated ALT.

**Table 4:** Odds ratio (95% confidence interval) of elevated ALT across tertiles of unhealthy foods: Tehran Lipid and Glucose Study

	Tertile 1	Tertile 2	Tertile 3	p for trend	Continues
Fast-food (%kcal/week)	*< 3.16*	*3.16–11.38*	*> 11.39*		
Crude	1.00	1.32 (0.82–2.11)	2.70 (1.68–4.34)	0.001	1.33 (1.09–1.63)
Adjusted model	1.00	1.41 (0.82–2.42)	2.74 (1.57–4.77)	0.001	1.02 (1.00–1.04)
Soft drink (%kcal/week)	*< 1.50*	*1.50–5.68*	*> 5.69*		
Crude	1.00	1.00 (0.63–1.60)	1.44 (0.90–2.29)	0.124	1.35 (1.10–1.66)
Adjusted model	1.00	0.71 (0.42–1.20)	0.93 (0.54–1.60)	0.798	1.02 (1.00–1.05)
Sweet snack (%kcal/week)	*< 13.48*	*13.48–30.42*	*> 30.43*		
Crude	1.00	0.95 (0.59–1.50)	1.28 (0.81–2.04)	0.291	1.18 (0.97–1.43)
Adjusted model	1.00	0.80 (0.48–1.34)	1.09 (0.64–1.85)	0.768	1.00 (1.00–1.01)
Salty snack (%kcal/week)	*< 3.08*	*3.08–11.66*	*> 11.67*		
Crude	1.00	1.54 (0.97–2.46)	1.54 (0.97–2.46)	0.069	1.02 (0.84–1.23)
Adjusted model	1.00	1.44 (0.85–2.42)	1.23 (0.71–2.14)	0.468	1.00 (0.98–1.01)

Logistic regression model was used (adjusted for sex, age, body mass index, diabetes status)

Elevated ALT was considered as values > 12 (U/L)

## Discussion

The results of our study indicated that higher energy intakes from fast foods were associated with an elevated serum levels of ALT and ALT to AST ratio in adults. Participants who had more energy intakes from fast foods (> 11.39% kcal/week) had more than two folds increased risk of elevated ALT (values ≥12 U/L), as a predictor of NAFLD. Furthermore, higher intake of fast foods was accompanied with more than three folds increased odds of elevated ALT to AST ratio (values ≥0.62). Other unhealthy foods, including soft drinks, sweet and salty snacks, were not associated with elevated ALT and ALT to AST ratio. Although the effect of diet on NAFLD and some liver enzymes was investigated in previous studies, to the best of our knowledge this is the first study to assess the association of unhealthy foods and ALT to AST ratio, as a clinical prognostic feature of fatty liver.

A growing number of evidences suggest that diet plays a key role in the development of NAFLD. One prospective cohort study in adolescents showed that Western dietary pattern including high consumption of soft drinks, sauces and dressings, was significantly associated with increased development of NAFLD after 3 years of follow-up [27]. Another observational studies also revealed that subjects with NAFLD had higher consumption of meat and fast foods [35] and exceeded energy and saturated fat intakes [36]. Results from two studies conducted in China reported that Western dietary pattern, with high amount of refined grains, soft drinks and red meat, as well as “Animal food” dietary pattern, with high amount of meat and egg, were related to an increased risk of NAFLD [37, 38].

Despite the reports for the relation between dietary factors and NAFLD, whether dietary factors are related to elevated levels of aminotransferases are less documented. Abnormal levels of liver enzymes, particularly aminotransferases, are commonly used in clinical setting to diagnose liver dysfunctions [6]. ALT and ALT to AST ratio are two important prognostic features of NAFLD and metabolic disorders e.g. insulin resistance [7, 11, 12]. A prospective cohort study by Lee et al. showed that higher consumption of fast foods or sugar-sweetened beverages increased risk of elevated ALT [39]. Daily eating of at least two high-calorie fast-food-based meals resulted in pathologic elevated levels of ALT; most of the participants showed ALT above reference limits (women > 19 U/l, men > 30 U/l) after 4 weeks [40]. A positive association between sugar-sweetened beverages and ALT or AST was also shown in a recent prospective cohort study [41]. Every 1 cup/day increased consumption of sugar-sweetened beverages, resulted in elevated logarithm of serum ALT concentration by 0.079 U/L (95% CI 0.022,0.137) [41]. Our findings provide further evidence regarding contribution of dietary factors on development of liver disorders. Furthermore, current literature indicates that increase increased level of liver enzymes even within normal ranges, increased risk of metabolic syndrome and diabetes mellitus [42, 43]. To put our findings within the context of these studies, diet can accelerate fatty liver progression even prior to appearance of clinical signs, which is detectable by increased levels of liver enzymes.

There are some proposed mechanisms through which unhealthy foods contribute to development of elevated liver enzymes. Most commonly consumed unhealthy foods, such as fast foods, soft drinks, sweet and salty snacks, are energy-dense and nutrient-poor foods, and contain high levels of fats, saturated and trans-fats, refined sugars and salts [44–46]. High amounts of refined and high glycemic index carbohydrates in energy-dense unhealthy foods, as well as high amounts of sweeteners such as sucrose and fructose in soft drinks and sweet snacks, are contributing factors in elevated liver enzymes and liver disorders [1, 41, 47, 48]. Some evidences suggest that fructose intake could stimulate de novo lipogenesis and inhibit mitochondrial beta-oxidation of fats [49–51], which leads to increased liver fat content and elevated liver enzymes levels. Unhealthy food intakes are also related to lower intakes of essential nutrients (e.g. fiber, protein, B vitamins) which are required for normal liver function [45, 52].

In the present study, the mean calorie intake from sweet and salty snacks were higher than other unhealthy foods (698 ± 758 kcal/week and 292 ± 469 kcal/week, respectively), but it was lower than per capita mean calorie intake from snacks in US adults (516 kcal/d in adults aged 19–29 y, 484 kcal/d in adults aged 30-59y) [53]. A null association between sweet or salty snack and elevated liver enzymes in our study, in contrast to previous studies, may relate to differences in amount of snacks consumption and mean calorie intake from snacks in different populations, or different definitions of sweet and salty snacks in studies, as well as various constituents in snacks and different preparation methods.

The current study had some limitations that should be considered; we used FFQ for dietary assessment which has some disadvantages including low accuracy due to recall bias, inaccurate estimation, under or over reports, and inherent limitation to capture eating habits. Also, we used the USDA food composition table to analyze energy and nutrient content of foods, rather than a complete Iranian table. Moreover, it was a cross sectional study which could not reveal any cause and effect relationship between unhealthy foods and liver enzymes. Inability to define a specific and reliable cut-off for elevated liver enzymes in relation to NAFLD, is also consider as a limitation.

## Conclusions

In conclusion, we provided further evidence to support previous investigations regarding adverse effects of fast foods consumption on liver function. An elevated serum concentration of ALT and ALT to AST ratio was observed in subjects who consumed more energy from energy-dense, poor-nutrients fast foods. There was no significant association between soft drinks, sweet and salty snacks with elevated levels of aminotransferases. More population-based studies with prospective design and long follow-up period in a larger sample size, are needed to confirm the effect of regular consumption of unhealthy foods on development of NAFLD and elevated liver enzymes.

## Data Availability

The datasets used and/or analyzed during the current study available from the corresponding author on reasonable request.

## Abbreviations

ALT: Alanine Transaminase
AST: Aspartate Transaminase
BMI: Body Mass Index
DBP: Diastolic Blood Pressure
LFT: Liver Function Test
NAFLD: Non-Alcoholic Fatty Liver Disease
SBP: Systolic Blood Pressure

## References

[1] Abid A, Taha O, Nseir W, Farah R, Grosovski M, Assy N (2009). Soft drink consumption is associated with fatty liver disease independent of metabolic syndrome. J Hepatol.

[2] Calzadilla Bertot L, Adams LA. The Natural Course of Non-Alcoholic Fatty Liver Disease. Int J Mol Sci. 2016;17(5):774-85. 10.3390/ijms17050774 PMC4881593 27213358

[3] Mohanty SR, Troy TN, Huo D, O'Brien BL, Jensen DM, Hart J (2009). Influence of ethnicity on histological differences in non-alcoholic fatty liver disease. J Hepatol.

[4] Moghaddasifar I, Lankarani KB, Moosazadeh M, Afshari M, Ghaemi A, Aliramezany M (2016). Prevalence of non-alcoholic fatty liver disease and its related factors in Iran. Int J Organ Transplant Med.

[5] Barzin M, Valizadeh M, Serahati S, Mahdavi M, Azizi F, Hosseinpanah F (2018). Overweight and Obesity: Findings from 20 Years of the Tehran Lipid and Glucose Study. Int J Endocrinol Metab.

[6] Adams LA, Angulo P, Lindor KD (2005). Nonalcoholic fatty liver disease. CMAJ.

[7] Dufour DR, Lott JA, Nolte FS, Gretch DR, Koff RS, Seeff LB (2000). Diagnosis and monitoring of hepatic injury. I. Performance characteristics of laboratory tests. Clin Chem.

[8] Martin-Rodriguez JL, Gonzalez-Cantero J, Gonzalez-Cantero A, Arrebola JP, Gonzalez-Calvin JL (2017). Diagnostic accuracy of serum alanine aminotransferase as biomarker for nonalcoholic fatty liver disease and insulin resistance in healthy subjects, using 3T MR spectroscopy. Medicine.

[9] Maximos M, Bril F, Portillo Sanchez P, Lomonaco R, Orsak B, Biernacki D (2015). The role of liver fat and insulin resistance as determinants of plasma aminotransferase elevation in nonalcoholic fatty liver disease. Hepatology.

[10] Schindhelm RK, Diamant M, Dekker JM, Tushuizen ME, Teerlink T, Heine RJ (2006). Alanine aminotransferase as a marker of non-alcoholic fatty liver disease in relation to type 2 diabetes mellitus and cardiovascular disease. Diabetes Metab Res Rev.

[11] Nanji AA, French SW, Freeman JB (1986). Serum alanine aminotransferase to aspartate aminotransferase ratio and degree of fatty liver in morbidly obese patients. Enzyme.

[12] Zhao L, Cheng J, Chen Y, Li Q, Han B, Chen Y (2017). Serum alanine aminotransferase/aspartate aminotransferase ratio is one of the best markers of insulin resistance in the Chinese population. Nutr Metab.

[13] Hanley AJ, Wagenknecht LE, Festa A, D'Agostino RB, Haffner SM (2007). Alanine aminotransferase and directly measured insulin sensitivity in a multiethnic cohort: the insulin resistance atherosclerosis study. Diabetes Care.

[14] Homsanit M, Sanguankeo A, Upala S, Udol K (2012). Abnormal liver enzymes in Thai patients with metabolic syndromes. J Med Assoc Thail.

[15] Karmen A, Wroblewski F, Ladue JS (1955). Transaminase activity in human blood. J Clin Invest.

[16] Piton A, Poynard T, Imbert-Bismut F, Khalil L, Delattre J, Pelissier E (1998). Factors associated with serum alanine transaminase activity in healthy subjects: consequences for the definition of normal values, for selection of blood donors, and for patients with chronic hepatitis C. MULTIVIRC Group. Hepatology.

[17] Prati D, Taioli E, Zanella A, Della Torre E, Butelli S, Del Vecchio E (2002). Updated definitions of healthy ranges for serum alanine aminotransferase levels. Ann Intern Med.

[18] Gharipour M, Sarrafzadegan N, Sadeghi M, Andalib E, Talaie M, Shafie D (2013). Predictors of metabolic syndrome in the Iranian population: waist circumference, body mass index, or waist to hip ratio?. Cholesterol.

[19] Jamali R, Pourshams A, Amini S, Deyhim MR, Rezvan H, Malekzadeh R (2008). The upper normal limit of serum alanine aminotransferase in Golestan Province, Northeast Iran. Arch Iran Med.

[20] Kabir A, Pourshams A, Khoshnia M, Malekzadeh F (2013). Normal limit for serum alanine aminotransferase level and distribution of metabolic factors in old population of Kalaleh, Iran. Hepat Mon.

[21] Neuman MG, Cohen LB, Nanau RM (2014). Biomarkers in nonalcoholic fatty liver disease. Can J Gastroenterol Hepatol.

[22] Mirmiran P, Amirhamidi Z, Ejtahed H-S, Bahadoran Z, Azizi F (2017). Relationship between diet and non-alcoholic fatty liver disease: a review article. Iran J Public Health.

[23] Bahadoran Z, Mirmiran P, Hosseini-Esfahani F, Azizi F (2013). Fast food consumption and the risk of metabolic syndrome after 3-years of follow-up: Tehran lipid and glucose study. Eur J Clin Nutr.

[24] Mirmiran P, Bahadoran Z, Delshad H, Azizi F (2014). Effects of energy-dense nutrient-poor snacks on the incidence of metabolic syndrome: a prospective approach in Tehran Lipid and Glucose Study. Nutrition.

[25] Bahadoran Z, Mirmiran P, Azizi F (2015). Undesirable Cardiometabolic outcomes of fast-food patterns. Iran J Public Health.

[26] Bahadoran Z, Mirmiran P, Azizi F (2016). Fast food pattern and Cardiometabolic disorders: a review of current studies. Health Promot Perspect.

[27] Oddy WH, Herbison CE, Jacoby P, Ambrosini GL, O'Sullivan TA, Ayonrinde OT (2013). The Western dietary pattern is prospectively associated with nonalcoholic fatty liver disease in adolescence. Am J Gastroenterol.

[28] Trovato FM, Martines GF, Catalano D (2018). Addressing Western dietary pattern in obesity and NAFLD. Nutrire.

[29] Azizi F, Ghanbarian A, Momenan AA, Hadaegh F, Mirmiran P, Hedayati M (2009). Prevention of non-communicable disease in a population in nutrition transition: Tehran lipid and glucose study phase II. Trials.

[30] Azizi F, Rahmani M, Emami H, Mirmiran P, Hajipour R, Madjid M (2002). Cardiovascular risk factors in an Iranian urban population: Tehran lipid and glucose study (phase 1). Soz Praventivmed.

[31] Askari S, Asghari G, Ghanbarian A, Khazan M, Alamdari S, Azizi F (2014). Seasonal variations of blood pressure in adults: Tehran lipid and glucose study. Arch Iran Med.

[32] Shim J-S, Oh K, Kim HC (2014). Dietary assessment methods in epidemiologic studies. Epidemiol Health.

[33] Hosseini-Esfahani F, Jessri M, Mirmiran P, Bastan S, Azizi F (2010). Adherence to dietary recommendations and risk of metabolic syndrome: Tehran lipid and glucose study. Metab Clin Exp.

[34] Mirmiran P, Esfahani FH, Mehrabi Y, Hedayati M, Azizi F (2010). Reliability and relative validity of an FFQ for nutrients in the Tehran lipid and glucose study. Public Health Nutr.

[35] Fakhoury-Sayegh N, Younes H, Heraoui G, Sayegh R. Nutritional Profile and Dietary Patterns of Lebanese Non-Alcoholic Fatty Liver Disease Patients: A Case-Control Study. Nutrients. 2017;9(11):1245-61. 10.3390/nu9111245 PMC5707717 29135945

[36] Ferolla SM, Ferrari TCA, Lima MLP, Tavares-Jr WC, Couto OFM, Reis TO (2013). Dietary patterns in Brazilian patients with non-alcoholic fatty liver disease: a cross-sectional study. Clinics.

[37] Liu X, Peng Y, Chen S, Sun Q (2018). An observational study on the association between major dietary patterns and non-alcoholic fatty liver disease in Chinese adolescents. Medicine.

[38] Yang CQ, Shu L, Wang S, Wang JJ, Zhou Y, Xuan YJ (2015). Dietary patterns modulate the risk of non-alcoholic fatty liver disease in Chinese adults. Nutrients.

[39] Lee E-y, Choi HY, Cho H, Kim BH, Ki M (2018). Health behavior associated with liver enzymes among obese Korean adolescents, 2009–2014. PLoS One.

[40] Kechagias S, Ernersson A, Dahlqvist O, Lundberg P, Lindstrom T, Nystrom FH (2008). Fast-food-based hyper-alimentation can induce rapid and profound elevation of serum alanine aminotransferase in healthy subjects. Gut.

[41] Shimony MK, Schliep KC, Schisterman EF, Ahrens KA, Sjaarda LA, Rotman Y (2016). The relationship between sugar-sweetened beverages and liver enzymes among healthy premenopausal women: a prospective cohort study. Eur J Nutr.

[42] Nikniaz L, Nikniaz Z, Tabrizi JS, Sadeghi-Bazargani H, Farahbakhsh M (2018). Is within-normal range liver enzymes associated with metabolic syndrome in adults?. Clin Res Hepatol Gastroenterol.

[43] Oh HJ, Kim TH, Sohn YW, Kim YS, Oh YR, Cho EY (2011). Association of serum alanine aminotransferase and gamma-glutamyltransferase levels within the reference range with metabolic syndrome and nonalcoholic fatty liver disease. Korean J Hepatol.

[44] Bowman SA, Gortmaker SL, Ebbeling CB, Pereira MA, Ludwig DS (2004). Effects of fast-food consumption on energy intake and diet quality among children in a national household survey. Pediatrics.

[45] Datar A, Nicosia N (2012). Junk food in schools and childhood obesity. J Policy Anal Manage.

[46] Ovaskainen ML, Reinivuo H, Tapanainen H, Hannila ML, Korhonen T, Pakkala H (2006). Snacks as an element of energy intake and food consumption. Eur J Clin Nutr.

[47] Assy N, Nasser G, Kamayse I, Nseir W, Beniashvili Z, Djibre A (2008). Soft drink consumption linked with fatty liver in the absence of traditional risk factors. Can J Gastroenterol.

[48] Sevastianova K, Santos A, Kotronen A, Hakkarainen A, Makkonen J, Silander K (2012). Effect of short-term carbohydrate overfeeding and long-term weight loss on liver fat in overweight humans. Am J Clin Nutr.

[49] Jensen T, Abdelmalek MF, Sullivan S, Nadeau KJ, Green M, Roncal C (2018). Fructose and sugar: a major mediator of nonalcoholic fatty liver disease. J Hepatol.

[50] Lim JS, Mietus-Snyder M, Valente A, Schwarz JM, Lustig RH (2010). The role of fructose in the pathogenesis of NAFLD and the metabolic syndrome. Nat Rev Gastroenterol Hepatol.

[51] Softic S, Cohen DE, Kahn CR (2016). Role of dietary fructose and hepatic De novo Lipogenesis in fatty liver disease. Dig Dis Sci.

[52] Tapsell LC, Neale EP, Satija A, Hu FB (2016). Foods, nutrients, and dietary patterns: interconnections and implications for dietary guidelines. Adv Nutr.

[53] Dunford EK, Popkin BM (2017). Disparities in snacking trends in US adults over a 35 year period from 1977 to 2012. Nutrients.

